# Associations Among Piglet Characteristics, Farrowing Kinetics, and Stillbirth Risk in Hyperprolific Sows

**DOI:** 10.3390/ani16142223

**Published:** 2026-07-17

**Authors:** Chananchida Mueansree, Phubet Satsook, Napatsorn Kamnaray, Nitikan Nikhomjit, Thanakorn Jongprasert, Niratchaporn Faksongsakul, Suppachok Taveekaikun, Nitipong Homwong

**Affiliations:** 1 Laboratory of Swine Science, Department of Animal Science, Faculty of Agriculture at Kamphaeng Saen, Kasetsart University Kamphaeng Saen Campus, Nakhon Pathom 73140, Thailand; chananchida.m@ku.th (C.M.); phubet.sat@ku.th (P.S.); napatsorn.kam@ku.th (N.K.); nitikan.n@ku.th (N.N.); thanakorn.jon@ku.th (T.J.); niratchaporn.f@ku.th (N.F.); 2 National Swine Research and Training Center, Faculty of Agriculture at Kamphaeng Saen, Kasetsart University Kamphaeng Saen Campus, Nakhon Pathom 73140, Thailand; 3 Papon Farm Research Station, Chombeung, Ratchaburi 70150, Thailand; suppachok3151@gmail.com

**Keywords:** birth, interval, weight, order, piglets, stillborn, survival

## Abstract

Stillbirth remains a major cause of piglet loss in hyperprolific sow herds. This observational study investigated the effects of farrowing kinetics, including birth weight (BW), birth order (BO), and expulsion interval (EI), on stillbirth risk. Data from 117 crossbred sows between Landrace sires and Yorkshire dams and 1726 piglets were analyzed using linear mixed models (LMM) and generalized linear mixed models (GLMM), with sow included as a random effect to account for litter clustering. BW, BO, time of farrowing (TF), and EI were categorized into three (BWG, low, medium, high), four (BOQ, Q1–Q4), four (H1–H4), and seven (≤5, 6–10, 11–15, 16–20, 21–30, 31–45, and >45 min) groups, respectively. BWG were not associated with BO (*p* = 0.22) nor with EI (*p* = 0.49). Univariate GLMM analysis showed that stillbirth risk increased across BOQ, with Q4 piglets exhibiting a higher stillbirth rate than Q1 piglets (9.1% vs. 1.6%; *p* < 0.01), whereas TF and EI groups were not associated with stillbirth risk (*p* > 0.05). In the multivariable GLMM, lower BW (OR = 0.49 per 0.25 kg increase; *p* < 0.001), longer cumulative expulsion interval (CEI) (OR = 1.71 per 60 min increase; *p* < 0.001), and greater within-litter birth weight variation (BWCV; OR = 1.51 per 10% increase; *p* = 0.077) were associated with an increased risk of stillbirth. In contrast, BOQ, EI group, litter size (LS), and TF were not retained in the final model. Reverse Kaplan–Meier survival analysis demonstrated a progressive ascend in the probability of being stillborn throughout the farrowing process (*p* < 0.01), with a median stillborn time of 450 min. These findings indicate that prolonged cumulative farrowing duration, rather than individual birth intervals or birth sequence alone, is the primary farrowing-related determinant of stillbirth risk. These findings suggest that reducing CEI, providing timely assistance to late-born piglets and lowering BWCV may help decrease stillbirth losses in hyperprolific sow herds.

## Simple Summary

Stillbirth during farrowing remains a major economic and animal welfare concern in modern hyperprolific sow production systems. This study evaluated data from 117 crossbred sows between Landrace sires and Yorkshire dams and 1726 piglets to identify factors associated with stillbirth, including birth weight, birth order, expulsion interval, sow parity, litter size, and time of farrowing. Late-born piglets were more likely to be stillborn than earlier-born littermates. Birth order itself was not associated with birth weight. The risk of stillbirth increased progressively as cumulative expulsion interval, so-called farrowing duration, became longer, indicating that prolonged cumulative exposure to the farrowing process poses a greater threat to piglet survival. Low birth weight and greater variation in piglet size within a litter were also important risk factors. These findings suggest that reducing total farrowing duration, providing timely assistance to late-born piglets and lowering variation in piglet size within a litter may help decrease stillbirth losses in hyperprolific sow herds.

## 1. Introduction

Stillbirth is a major component of perinatal mortality in hyperprolific sow herds, accounting for approximately 5–10% of all piglets born [1,2]. Most stillborn piglets die during parturition; this is therefore potentially applicable to management of the farrowing process [3]. These losses not only compromise animal welfare but also reduce the economic sustainability of commercial swine production systems [4]. Several piglet and sow characteristics influenced stillbirth. Prenatal conditions, particularly intrauterine growth restriction (IUGR), contribute to low birth weight (BW), and increased within-litter birth weight variation (BWCV) is associated with reduced intrapartum viability [5,6]. Furthermore, larger litters are often associated with increased BWCV because of greater intrauterine competition for nutrients and uterine space [1,7,8,9]. Male piglets have been additionally reported to carry a higher stillbirth probability than females [10]. Sow-related factors also influence piglet survival. Higher parity has been associated with increased preweaning mortality, whereas reduced backfat thickness during late gestation has been linked to a greater incidence of stillbirth [7,8,9]. Obstetric management is influential. Administration of oxytocin during the expulsive phase can provoke tetanic uterine contractions and premature placental separation, aggravate fetal hypoxia, and increase stillbirth risk [11,12]. Obstetric intervention is also associated with elevated stillbirth odds, particularly applicable when dystocia is already present [10].

Although piglet characteristics describe the biological condition of piglets at birth, farrowing kinetics characterize the temporal dynamics of the farrowing process. Farrowing kinetics can be quantified using three interrelated parameters: birth order (BO), expulsion interval (EI), and cumulative expulsion interval (CEI). BO refers to the sequence in which piglets are delivered and may influence survival through prolonged exposure to uterine contractions, progressive fetal hypoxia, depletion of energy reserves, and reduced uterine contractility [5,13,14,15,16]. Consequently, late-born piglets often exhibit higher mortality rates than their earlier-born littermates. EI, defined as the time between the birth of two consecutive piglets, is another important determinant of piglet survival. Under normal conditions, EI ranges from approximately 10 to 20 min [2]. However, prolonged EI may compromise oxygen delivery to the fetus because of premature placental separation or umbilical cord compression, thereby increasing the risk of intrapartum asphyxia [17,18]. Previous studies have demonstrated a curvilinear relationship between EI and stillbirth risk [19]. CEI, defined as the elapsed time from the birth of the first piglet to that of a given piglet, reflects the cumulative exposure of fetuses to the farrowing process. Prolonged CEI has detrimental effects on piglet viability through progressive maternal fatigue, reduced myometrial efficiency, and increasing fetal hypoxia. Farrowing durations exceeding 300 min have been identified as an important risk factor for reduced offspring viability [20,21]. The pathophysiology of intrapartum stillbirth is closely linked to prolonged farrowing. Repeated uterine contractions progressively reduce uteroplacental blood flow, while rupture or compression of the umbilical cord further limits oxygen delivery to the fetus. These events result in anaerobic metabolism, lactate accumulation, metabolic acidosis, and ultimately fetal death [3,19]. Therefore, piglets born late in the farrowing sequence, characterized by high BO and prolonged CEI, are particularly vulnerable to intrapartum mortality [3].

Despite the biological interdependence of BO, EI, and CEI, most previous studies have examined these variables separately and evaluated only one parameter at a time [19,22]. These cannot separate their individual contributions to stillbirth risk and may misattribute to one parameter an effect that is in fact driven by another. Furthermore, CEI is mathematically derived from the cumulative sum of individual EIs, and both CEI and EI are inherently related to BO. Several studies have proposed threshold values for increased mortality risk, including EI greater than 45 min [17] or greater than 90 min [19], and total farrowing duration exceeding 300 min [20]. However, converting continuous farrowing variables into categorical thresholds may obscure important gradients of risk and reduce statistical power [12,23], but this is beneficial for avoiding a linear relationship assumption between independent variables [24]. In addition, many previous investigations have analyzed farrowing outcomes at the litter level using analysis of variance or correlation approaches [5,25], resulting in inconsistent findings regarding the importance of CEI as a predictor of piglet survival [12,21].

To date, few studies have simultaneously evaluated BO, EI, and CEI within a single piglet-level analytical framework while accounting for both piglet and sow characteristics. Therefore, the present study incorporated farrowing kinetics (BO, EI, and CEI) together with piglet and spontaneously farrowing sow characteristics into a unified piglet-level model to predict stillbirth probability under tropical commercial production conditions. We hypothesized that differences in piglet characteristics and farrowing kinetics contribute independently to stillbirth risk. Accordingly, the objective of this study was to determine the effects of piglet characteristics and farrowing kinetics on stillbirth risk in a commercial swine herd.

## 2. Materials and Methods

### 2.1. Experimental Location and Ethical Approval

The study was conducted in a commercial swine breeding herd located in Chom Bueng District, Ratchaburi Province, Thailand. The herd was housed in environmentally controlled facilities equipped with an evaporative cooling system and automated climate-control and ventilation management (BlueControl Climate, SKOV A/S, Glyngore, Denmark). Animal handling and experimental procedures were approved by the Kasetsart University Animal Care and Use Committee (Approval No. ACKU68-AGK-079) and complied with institutional guidelines for animal welfare and ethical research practices.

### 2.2. Animals and Study Design

Data were collected from 117 crossbred sows between Landrace sires and Yorkshire dams with parity ranging from 1 to 5, yielding a total of 1778 piglets. This observational study investigated the associations between piglet characteristics, sow characteristics, and farrowing kinetics with stillbirth risk at the piglet level. Piglet survival status (live-born or stillborn) was considered the primary outcome variable, whereas BO, BW, EI, CEI, litter size (LS), parity, and time of farrowing (TF) were evaluated as explanatory variables.

### 2.3. Housing and Environmental Management

During gestation, sows were housed individually in gestation stalls (approximately 2.0 × 0.8 m) within environmentally controlled barns equipped with evaporative cooling systems. Ambient temperature and relative humidity were maintained at approximately 28–32 °C and 65–75%, respectively. On day 109 of gestation (approximately 7 days before the expected farrowing date), sows were transferred to individual farrowing crates (approximately 2.5 × 2.0 m). Each crate consisted of a concrete floor for the sow and a plastic-slatted creep area for piglets. The piglet creep area provided approximately 2 m^2^ of space and was equipped with a heated creep box containing a 200 W infrared lamp to maintain temperatures between 33 and 35 °C throughout the suckling period. Fresh water was available ad libitum to all sows through nipple drinkers during both gestation and lactation.

### 2.4. Nutritional Management

The gestation and lactation diets were formulated using FeedLIVE^®^ Version 1.51 (Live Informatics Co., Ltd., Nonthaburi, Thailand) to meet or exceed the nutrient requirements for breeding sows [26]. During gestation, sows were fed a diet containing 14.0% crude protein, 2900 kcal/kg metabolizable energy (ME), 10.9% crude fiber, and 0.80% total lysine. Feed was provided at 1.8–2.0 kg/day during early and mid-gestation and was gradually increased to 3.6–4.2 kg/day during the final four weeks before farrowing. Following farrowing, sows received a lactation diet containing 18.0% crude protein, 3200 kcal/kg ME, 8.76% crude fiber, and 0.93% total lysine. Feed allowance was restricted to 3.0 kg/day on the first day of lactation and then progressively increased until ad libitum intake was achieved by day 10 of lactation. Fresh water was available ad libitum throughout the study period.

### 2.5. Farrowing Management and Supervision

Sows were allowed to farrow spontaneously under routine farm management conditions. Only sows that farrowed without the administration of oxytocin or obstetrical intervention were included in the study. All other standard husbandry procedures were performed by trained farm personnel. After the birth of the last piglet, each sow received an intravenous infusion of calcium borogluconate solution via an ear vein to prevent hypocalcemia. These procedures were part of the farm’s standard management protocol and were applied uniformly to all sows. The infusion consisted of 30 mL calcium borogluconate diluted in 1 L of a 5% dextrose and 0.45% sodium chloride solution and was administered over approximately 10–15 min. Following complete expulsion of the placentas, sows were treated with flunixin meglumine (2 mL, intramuscular injection; Norbrook Laboratories Limited, Newry, Northern Ireland) as a non-steroidal anti-inflammatory drug (NSAID) to support post-farrowing recovery.

### 2.6. Neonatal Piglet Health Care

Immediately after birth, placental fluids and membranes were removed from each piglet, and the body surface was dried using a commercial drying powder (Inner Mongolia Hezhengmei Biology Science and Technology Co., Ltd., Chifeng, China). Each piglet was individually identified by a unique number marked on the dorsal surface with a permanent marker and weighed using a digital scale (precision ± 0.01 kg; VT TAN SCALE Co., Ltd., Nakhon Pathom, Thailand). Live-born piglets were assisted in obtaining colostrum within 30 min after birth. Cross-fostering was performed only after completion of data collection related to farrowing and neonatal survival and was performed at 48 h post-farrowing to standardize litter sizes to 14–15 piglets per sow. Piglets from larger litters were preferentially selected for fostering to reduce within-litter birth weight variation and improve litter uniformity. Routine piglet management procedures included tooth grinding; oral administration of toltrazuril (Baycox^®^ 5%; Elanco Animal Health, Greenfield, IN, USA); tail docking; and intramuscular injection of iron dextran (2 mL; 200 mg Fe; Uniferon^®^; Pharmacosmos A/S, Holbæk, Denmark) at 3 days of age. Male piglets were surgically castrated at 7 days of age. All procedures were performed by trained farm personnel according to standard farm management protocols.

### 2.7. Data Collection

All reproductive performance records were digitally maintained using PigLIVE^®^ Version 4.0 (Live Informatics Co., Ltd., Nonthaburi, Thailand). Sow-level variables recorded for each farrowing included parity, birth time of each piglet, total farrowing duration (defined as the interval from the birth of the first piglet to the birth of the last piglet), total number born (TB), number born alive (BA), number of stillborn piglets (SB), number of mummified fetuses, and total litter birth weight (calculated as the sum of individual live-born piglet birth weights).

A stillborn piglet was defined as a fully developed piglet that did not breathe at birth and exhibited intact periople on the hooves, indicating death occurred before or during parturition [27]. Mummified fetuses were recorded but excluded from all subsequent statistical analyses. Piglet-level variables included BO; sequential position in the farrowing sequence, TF (h:min), BW, sex, and vital status at birth (live-born, stillborn, or mummified). These data were used to evaluate the relationships among piglet characteristics, farrowing kinetics, and stillbirth risk.

BO was assigned within each litter according to the recorded birth times of individual piglets. EI was defined as the time elapsed between the births of two consecutive piglets and was calculated as the time elapsed between the births of two consecutive piglets, whereas CEI was calculated as the elapsed time from the birth of the first piglet to the birth of each subsequent piglet and was calculated as the cumulative sum of all preceding EIs. Consequently, CEI represented the cumulative duration of exposure to the farrowing process experienced by each piglet.

### 2.8. Statistical Analysis

#### 2.8.1. Exclusion Criteria and Data Preprocessing

When two piglets were recorded with identical birth times, the corresponding interval was divided equally between them. Sows were excluded from the analysis if they had more than three piglets with identical recorded birth times, a maximum expulsion interval exceeding 150 min, or more than five mummified fetuses. Mummified fetuses were excluded from all statistical analyses. After data screening, 117 sows and 1778 piglets were recorded, excluding 52 mummified fetuses. A total of 1726 piglets remained for statistical analysis. The distributions of TF and EI were assessed for normality using the Kolmogorov–Smirnov test. To avoid assuming a linear relationship between these variables and stillbirth risk, both variables were categorized prior to analysis [24]. TF was classified into four 6 h periods: H1 (00:00–05:59 h), H2 (06:00–11:59 h), H3 (12:00–17:59 h), and H4 (18:00–23:59 h). EI was categorized into seven groups: ≤5, 6–10, 11–15, 16–20, 21–30, 31–45, and >45 min.

Sows were stratified into three parity groups: parity 1 (primiparous), parity 2, and parity 3–5 (multiparous). BW was categorized into three groups (BWG; low, medium, and high), each containing approximately one-third of the piglets. Within-litter BW coefficient of variation (BWCV) was calculated as: BWCV (%) = (SD/Mean BW) × 100 and subsequently categorized into quartiles (BWCV: Q1–Q4).

BO was also categorized into quartiles (BOQ) according to each piglet’s relative position within the litter: Q1 (first 25% of piglets born), Q2 (26–50%), Q3 (51–75%), and Q4 (76–100%).

#### 2.8.2. Descriptive Statistics

Sow and piglet level characteristics were summarized as mean ± SD with min to max, and the distribution of farrowing duration was visualized as a frequency histogram.

#### 2.8.3. Influence of BW-Categorized Group on BO and EI

BO and EI were analyzed as a continuous variable. The effects of BW category and parity group on BO and EI were analyzed using linear mixed models (LMM), with BW category and parity group fitted as fixed effects and sow fitted as a random effect. This approach accounted for the hierarchical structure of piglets nested within sows. The model was specified as follows:

Y*_ijkl_* = μ + β_1_BWG*_i_* + β_2_Parity*_k_* + Sow*_j_* + e*_ijkl_* (1)

where Y*_ijkl_* is the BO or EI of piglet *l* from BW category *i* from sow *j* with parity *k*; μ the overall mean; BWG*_i_* is fixed effect of BW category *i* (*i* = 1, 2, 3); Parity*_k_* is fixed effect of parity group *k* (*k* = 1, 2, 3–5); Sow*_j_* ~ N(0, σ^2^*_sow_*) is random effect of sow *j*, and e*_ijkl_* ~ N(0, σ^2^) is the residual error.

#### 2.8.4. Stillbirth Analysis via Generalized Linear Mixed Models with Binomial Distribution and Logit Link Function

Piglet stillbirth status was analyzed as a binary outcome variable, where 0 = live-born and 1 = stillborn. The associations between stillbirth risk and farrowing kinetics, including TF, BO, and EI, were evaluated using generalized linear mixed models (GLMM) with a binomial distribution and logit link function. Since the observed number of stillborn piglets corresponding to TF was near zero and the expected number cannot be lower than zero, a quasi-binomial likelihood method was used to estimate the mean-variance relationship and its confidence interval [28,29]. In all models, a sow was included as a random effect to account for the clustering of piglets within litters. The general model structure was as follows:

logit(p*_ijl_*) = μ + β_1_X*_ij_* + Sow*_j_* + e*_ijl_* (2)

where p*_ijl_* is the probability that piglet *l* from sow *j* is stillborn; μ is intercept term (overall mean) while β_1_ is logistic regression coefficient. X*_il_* is fixed effect of the predictor variable *i* (time of farrowing: *i* = 1–4; BOQ: *i* = 1–4, and EI groups: *i* = 1–7, Sow*_j_* ~ N(0, σ^2^*_sow_*) is the random intercept of sow *k*, and e*_ijl_* ~ N(0, π^2^/3) is the residual error of binomial distribution and logit link function [24].

Original EI (treated as a continuous variable) was further evaluated for potential non-linear associations with stillbirth by comparing models containing linear, quadratic, and cubic terms. Model fits were assessed using the Akaike Information Criterion (AIC), and the model with the lowest AIC was selected. CEI was summarized descriptively.

To investigate the multivariable associations between farrowing kinetics, litter characteristics, and individual piglet characteristics with the likelihood of stillbirth, GLMM was constructed. Candidate fixed effects included BW, EI, CEI, BWCV, BOQ, parity group, LS, and TF. Continuous predictors (BW, CEI, and BWCV) were standardized to a mean of 0 and a standard deviation of 1 prior to analysis. Firstly, the full model was specified as follows:

logit(p*_ijkl_*) = β_0_ + β_1_BW*_ijk_* + β_2_EI*_ijk_* + β_3_CEI*_ijk_* + β_4_BWCV*_ij_* + β_5_BOQ*_ij_* +β_6_Parity*_k_* + β_7_LS*_ij_* +β_8_TF*_ij_* + Sow*_j_* + e*_ijkl_* (3)

where p*_jkl_* is the stillbirth probability of piglet *l* for *i* in sow *j* in parity *k*; *i* is fixed effect of the predictor variable; BW*_ijk_*, EI*_ijk_* and CEI*_ijk_* are piglet-level covariates; BWCV*_ij_*, BOQ*_ij_*, LS*_ij_* and TF*_ij_* are sow-level covariates; Parity*_k_* is parity group *k* (*k* = 1, 2, 3–5); Sow*_j_* ~ N(0, σ^2^*_sow_*) is the random intercept for sow *j*, and e*_ijkl_* ~ N(0, π^2^/3) is the residual error of binomial distribution and logit link function [24].

Then, models were compared using AIC, and the final model was selected based on the lowest AIC and biologically relevant covariate associated with stillbirth risk. Secondly, the final model was specified as follows:

logit(p*_jkl_*) = β_0_ + β_1_BW*_jkl_* + β_2_CEI*_jkl_* + β_3_BWCV*_k_* + β_4_Parity*_j_* + Sow*_k_* + e*_jkl_* (4)

where p*_jkl_* is the stillbirth probability of piglet *l* in sow *k* in parity *j*, BW*_jkl_* and CEI*_jkl_* are piglet-level covariates, BWCV*_k_* is sow-level covariates, Parity*_j_* is parity group *j* (*j* = 1, 2, 3–5), Sow*_k_* ~ N(0, σ^2^*_sow_*) is the random intercept for sow *k*, and e*_jkl_* ~ N(0, π^2^/3) is the residual error of binomial distribution and logit link function [24]. Odds ratios (ORs) were obtained by exponentiating the estimated regression coefficients and are reported with their corresponding 95% confidence intervals (95% CIs).

#### 2.8.5. Stillborn Probability at the Time of Farrowing

Stillborn probability was interpreted as the probability of a piglet being stillborn as CEI increased during farrowing. The probability throughout the farrowing process was estimated using the Reverse Kaplan–Meier method, with CEI as the time scale. Stillborn piglets were considered events, whereas the end of a farrowing event was treated as right-censored observations at their respective CEI values [30]. Differences in stillborn probability distributions were assessed using the log-rank test. Median stillborn time was defined as the CEI at which the estimated stillborn probability was at 0.50.

#### 2.8.6. Software and Packages

All statistical analyses were performed using R version 4.6 [31]. Data visualization was conducted using the “ggplot2” package [32]. Model assumptions, including normality, homoscedasticity, and linearity, were assessed using the “performance” package [33]. LMMs and GLMMs were fitted using the “lme4” package, with *p*-values calculated using the “pbkrtest” package [34]. Statistical significance was declared at *p* < 0.05. Pairwise comparisons among groups were performed using the “emmeans” package [35], with Tukey-adjusted multiple comparisons. Results are presented as estimated marginal means ± standard error (SE). Survival analyses were conducted using the “survival” package [36]. Kaplan–Meier survival curves were compared using the log-rank test, and statistical significance was defined as *p* < 0.05.

## 3. Results

### 3.1. Descriptive Statistics of Sow and Piglet Characteristics

**Figure 1:**
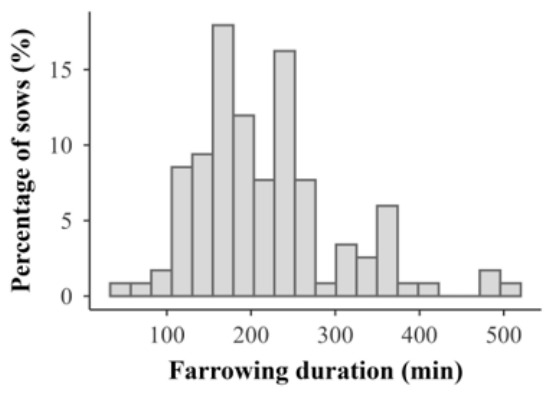
Percentage distribution of total farrowing duration in 117 Yorkshire × Landrace sows.

A total of 117 sows produced 1778 piglets, including 1726 live-born piglets, 104 stillborn piglets (6.0%), and 52 mummified fetuses (2.9%). Summary statistics are presented (Table 1). The CEI distribution was positively skewed, with 68.3% of litters completing farrowing within 100–300 min (Figure 1).

### 3.2. Effects of Birth-Weight Groups on Birth Order and Expulsion Interval

**Figure 2:**
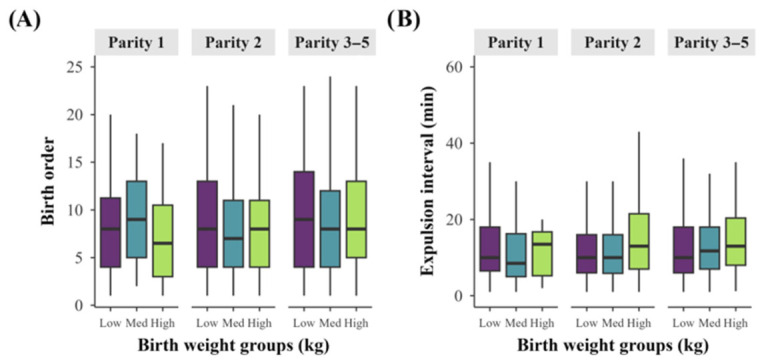
Effects of BW groups (Low: ≤ 1.25 kg; Med: 1.25 to 1.55 kg; High: ≥ 1.55 kg) on birth order (*p* = 0.22); (**A**) and expulsion interval (*p* = 0.49); (**B**) stratified by sow parity group (Parity 1, 2, and 3–5). The *p*-value was obtained from LMM with sow as a random effect.

BW was not associated with either BO (*p* = 0.22) or EI (*p* = 0.49). Across BWG, average BO ranged from 7.5 to 9.0 and did not differ within any parity group (Figure 2A). Similarly, mean EI ranged from 13.4 to 17.4 min and was not affected by BWG within any parity stratum (Figure 2B).

BO differed among parity groups (*p* = 0.04), with piglets born to parity 3–5 sows exhibiting higher mean BO values (8.5–9.0) than those born to parity 2 sows (7.5–8.0). EI also varied across BO quartiles (*p* < 0.001), decreasing as BO quartile increased. Piglets in the first BOQ (Q1) had longer EI values (18.2–21.7 min) compared with piglets in subsequent quartiles (Q2–Q4; 10.9–17.2 min).

### 3.3. Relationship Between Time of Day, Birth Order, and Expulsion Interval of Stillborn Piglets

**Figure 3:**
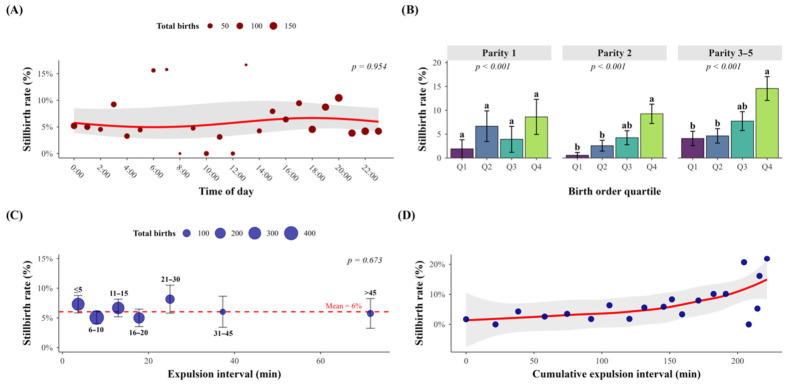
Relationships between stillborn rate and farrowing kinetics. (**A**) Stillborn rate by time of farrowing; point size indicates the number of piglets born in each interval. (**B**) Stillborn rate by BO quartile; ^a, b^ indicates significant differences within parity group. (**C**) Stillborn rate by EI category; point size indicates the number of births per category and dashed line represents the overall means (6%). (**D**) Stillborn rate by CEI, with 95% CI band.

The highest proportion of stillborn piglets was observed during the 06:00–08:00 period (20.5%), whereas the lowest proportion occurred during the 10:00–12:00 period (4.8%). However, obtained from a quasi-binomial likelihood method, stillbirth rate was not significantly associated with time-of-farrowing group within the 24 h period (*p* = 0.28; Figure 3A).

BOQ was significantly associated with stillbirth rate (*p* < 0.001; Figure 3B). Piglets born in the fourth BO quartile (Q4) exhibited an approximately six-fold greater stillbirth rate than those born in the first quartile (Q1) (9.1% vs. 1.6%). No differences in stillbirth rate were observed among piglets born in Q1, Q2, and Q3.

EI was not associated with stillbirth rate (Figure 3C), whether modeled as a linear (*p* = 0.67), quadratic (*p* = 0.45), or cubic (*p* = 0.22) term. In contrast, stillbirth rate tended to increase with increasing CEI (Figure 3D). Piglets born within the first 50 min of farrowing exhibited relatively low stillbirth rates (1.8–5.2%), whereas stillbirth rates increased markedly after 200 min of farrowing, ranging from 10.5% to 20.5%.

### 3.4. Risk Factors for Stillborn Piglets

Ten candidate GLMMs were compared using AIC (Table 2), with lower AIC values indicating better model fit. The AIC difference is small between Model 5 and Model 6. Therefore, Model 5 and Model 6 were selected as the candidate final models. Eventually, Model 6 was selected because it had a comparable AIC to the best-fitting model while retaining parity group, a biologically relevant covariate associated with stillbirth risk.

Model 6 was selected as the final model (AIC = 405.35), as it represents a biologically relevant covariate associated with stillbirth risk. The final model included BW, CEI, BWCV and parity group as fixed effects, with sow included as a random effect. Inclusion of EI, TF, and LS did not improve model fit, and these variables were therefore excluded from the final model.

BW was strongly associated with a reduced risk of stillbirth. For every 0.25 kg increase in BW, the odds of stillbirth decreased by approximately 51% (OR = 0.49, *p* < 0.001). In contrast, CEI was positively associated with stillbirth risk, with each additional 60 min of CEI increasing the odds of stillbirth by 71% (OR = 1.71, *p* < 0.001). Similarly, BWCV was positively associated with stillbirth risk, with each 10% increase in BWCV increasing the odds of stillbirth by 51% (OR = 1.51, *p* < 0.077) (Table 3).

### 3.5. Kaplan–Meier Survival Analysis

**Figure 4:**
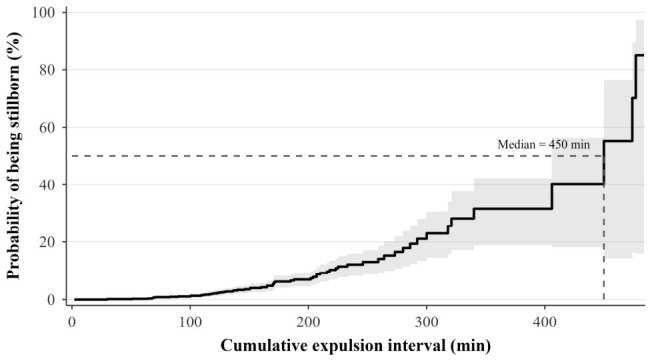
Reverse Kaplan–Meier survival probability curve through parturition process on cumulative expulsion interval. The solid line shows the estimated probability of being stillborn and the shaded band the 95% CI. Dashed lines indicate the median stillborn time (450 min).

The Reverse Kaplan–Meier survival curve indicated that the probability of piglets being stillborn increased progressively as CEI increased during farrowing (Figure 4). Estimated stillborn probabilities were 1.07% (95% CI 0.7–1.7), 4.1% (95% CI 3.1–5.3), and 5.6% (95% CI 4.4–7.1) at 100, 200, and 300 min of CEI, respectively. The median stillborn time was estimated at 450 min, indicating that the probability of being stillborn ascends to 50% after approximately 450 min (7.5 h) of farrowing.

## 4. Discussion

The hypothesis of the present study was that piglet characteristics and farrowing kinetics, BO, EI, and CEI, contribute differently to stillbirth risk. Most previous studies have examined BO, EI, and CEI separately, evaluating each parameter independently. However, these variables are intrinsically related because CEI is derived from the cumulative sum of individual EIs, making their independent interpretation challenging. To the best of our knowledge, this is the first study to simultaneously evaluate multiple farrowing kinetic parameters using integrated statistical models to identify the most relevant predictors of stillbirth. The results demonstrated that BW groups were not associated with either categorized BO or EI. Nevertheless, BW was strongly associated with stillbirth risk. Although late-born piglets (Q4) exhibited a greater risk of stillbirth than early-born piglets (Q1), this effect was largely explained by the longer CEI experienced by piglets born later in the farrowing sequence. Consequently, BW, CEI, and BWCV emerged as the primary predictors of stillbirth. These findings highlight the multifactorial nature of stillbirth and suggest that management strategies should focus on identifying and supporting low-BW and late-born piglets in hyperprolific sow production systems.

The present study found no association between BW groups and BO. Similarly, previous studies reported no effects of fetal sex or intrauterine position on BW [37]. In contrast, other studies have shown that fetuses located at the ovarian and cervical ends of the uterine horn tend to be heavier than those occupying central positions [37,38,39,40], suggesting that intrauterine location may influence fetal growth. Furthermore, several reports have demonstrated that piglets born later in the birth sequence tend to have lower BW and higher stillbirth rates [7,25]. BWCV also increases with parity and litter size because larger litters intensify intrauterine competition for nutrients and space [41]. This observation is consistent with the higher BWCV observed among Q4 piglets in the present study. In contrast, smaller litters in primiparous sows reduce intrauterine crowding and may promote more uniform fetal growth across uterine positions [8].

EI decreased from early to late BOQ, particularly in multiparous sows, reflecting physiological changes in myometrial contractility throughout parturition. Swine parturition progresses can explain the relationship between EI and BO. Q1 piglets are expelled before uterine contractions reach maximal efficiency, while coordinated myometrial activity is still being established under declining progesterone, rising estrogen and prostaglandin F_2α_, and upregulation of oxytocin receptors [42,43]. This corresponds to the longer EIs (18–20 min) observed in the present study. As parturition advances, stronger and more coordinated contractions, greater oxytocin responsiveness, and enhanced gap-junction formation facilitate fetal passage [11,43,44,45]. Consequently, piglets born later in the sequence (Q4) are expelled at shorter individual EIs (12–13 min). However, all sows in this study received an intravenous calcium borogluconate infusion after the birth of the first piglet as routine hypocalcemia prophylaxis. Although it was applied uniformly to all sows, calcium administration can influence uterine contractility and farrowing duration.

Although time of birth was not significantly associated with stillbirth, numerical differences were observed. The small numbers of spontaneously farrowing sows during the nighttime may affect statistical power. Stillbirth incidence was highest between 06:00 and 08:00 h (20.5%) and lowest between 10:00 and 12:00 h (4.8%). These findings suggest that farrowing supervision may influence productivity outcomes under commercial conditions. One possible explanation is the change of work shifts during early morning hours, which may reduce the level of monitoring and timely intervention during farrowing [27,46]. Environmental factors may also contribute, as low ambient temperatures and high relative humidity (>70%) can increase neonatal losses through hypothermia [47,48].

Stillbirth risk increased progressively across BO categories, particularly in multiparous sows, where Q4 piglets exhibited approximately a six-fold higher stillbirth rate than Q1 piglets. This finding is consistent with previous studies demonstrating greater mortality among late-born piglets [13,40]. Prolonged farrowing duration increases uterine fatigue, resulting in extended compression of late-born piglets within the birth canal [49]. Consequently, these piglets experience prolonged hypoxia and oxygen deprivation during parturition [3,16]. Delayed access to colostrum may further compromise postnatal survival of Q4 piglets [13,14].

Importantly, CEI rather than EI was retained in the final GLMM. This finding suggests that cumulative exposure to the farrowing process is more biologically relevant than the duration of any single interval between births. While individual EIs varied among piglets, the cumulative burden of repeated uterine contractions and progressive reductions in placental oxygen supply appear to be the major determinants of stillbirth risk. The average EI observed in the present study (15.83 min) was comparable to values reported in tropical commercial swine herds (14–16 min) [2,40] but shorter than the 20.6 min reported in litters with all piglets born alive [21].

The absence of an association between EI and stillbirth contrasts with previous studies reporting shorter EIs among live-born piglets compared with stillborn piglets [2] and studies suggesting that EIs exceeding 45 min substantially increase stillborn risk due to fetal oxygen deprivation [16]. These discrepancies likely reflect differences among studies in statistical methodology, LS, genetic background, individual farrowing dynamics, environmental conditions, and farrowing supervision protocols. Collectively, the present findings indicate that CEI provides a more robust measure of stillbirth risk than EI alone, particularly in hyperprolific sow populations.

The final GLMM identified BW as the strongest predictor of stillbirth. For every 0.25 kg increase in BW, the odds of stillbirth decreased by 51%, indicating that BW acted as a protective factor against stillbirth. The influence of BW on stillborn risk has been reported both before and during farrowing [2], and low-BW piglets have consistently been shown to be at a substantially greater risk of stillbirth [22]. Furthermore, low-BW piglets are more susceptible to intrapartum asphyxia because of limited glycogen reserves, lower blood pH, and elevated blood CO_2_ and lactate concentrations, which increase their vulnerability to hypoxia during uterine contractions [16,50]. BWCV also tended to increase the risk of stillbirth, with the odds increasing by 51% for every 10% increase in BWCV. This finding suggests that litter uniformity plays an important role in piglet stillbirth during parturition. High BWCV, which was most frequently observed among Q4 piglets and multiparous sows, reflects greater within-litter size heterogeneity, likely resulting from uneven uteroplacental nutrient supply and intrauterine crowding [8,41]. CEI was another significant risk factor. Each additional 60 min of farrowing increased the odds of stillbirth by 71%, corresponding to an approximately 2.92-fold increase after 120 min. Prolonged CEI likely reflects increasing fetal hypoxia associated with repeated uterine contractions and reduced placental blood flow during parturition. Importantly, BO and LS were not retained in the final GLMM, although BO was significantly associated with stillbirth in the univariate analysis. This suggests that the effects of BO and LS were largely mediated through CEI and BWCV, respectively. Consequently, CEI and BWCV may provide more comprehensive measures of stillborn risk because they capture the underlying biological consequences of both birth sequence and litter characteristics.

The Reverse Kaplan–Meier survival analysis demonstrated a progressive accumulation of stillborn risk as farrowing duration increased. Stillborn probability ascended from 1.07% at 100 min to 4.1% at 200 min and 5.6% at 300 min, with a median stillborn time of 450 min (7.5 h). This estimate closely agrees with a previous study reporting a median stillborn time of 450–500 min [21]. The consistency between studies suggests that the association between CEI and piglets being stillborn is robust and may be applicable across different genetic backgrounds. However, the curve highlights the adverse effects associated with prolonged farrowing duration but does not provide a definitive time point at which intervention should be initiated. Consequently, management strategies should focus on reducing overall farrowing duration rather than applying a fixed intervention threshold.

## 5. Conclusions

Late-born piglets were more likely to be stillborn than their earlier-born littermates. The major risk factors were prolonged cumulative expulsion interval (CEI), low individual BW, and greater BWCV, whereas time of day, birth interval, and LS showed no significant effects. These findings demonstrate the multifactorial nature of stillbirth and suggest that reducing CEI, providing timely assistance for late births and lowering BWCV may help decrease stillborn losses in hyperprolific sow herds.

## Figures and Tables

**Table 1:** Descriptive statistics of sow reproductive performance and newborn piglet characteristics.

Variable	P1	P2	P3 to P5	Mean ± SD	Min to Max
Sow-level data (*n* = 117)					
Number, head	15	56	46	-	-
Parity	1	2	3.74 ± 0.88	2.56 ± 1.15	1–5
TB, head	14.70 ± 2.55	13.60 ± 4.31	16.20 ± 3.77	14.75 ±4.08	4–28
NBA, head	13.90 ± 2.55	13.00 ± 3.94	14.90 ± 3.42	13.86 ± 3.67	4–24
SB, head	0.80 ± 1.37	0.59 ± 1.02	1.28 ± 1.22	0.89 ± 1.19	0–5
MM, head	0.80 ± 1.08	0.43 ± 0.89	0.35 ± 0.64	0.44 ± 0.84	0–3
CEI, min	193.13 ± 61.96	205.79 ± 85.59	240.01 ± 91.00	217.62 ± 86.10	45–508
LW, kg	18.78 ± 3.09	19.58 ± 4.65	21.09 ± 5.00	20.09 ±4.69	6.90–32.7
Piglet-level data (*n* = 1726)					
Number	221	760	745	-	-
BW, kg	1.27 ± 0.29	1.44 ± 0.36	1.35 ± 0.35	1.37 ± 0.38	0.35–2.50
EI, min	13.60 ± 13.90	16.50 ± 16.60	16.10 ± 18.40	15.83 ± 17.02	0–178

TB: Total number of piglets born per litter; NBA: Number of piglets born alive per litter; SB: Number of stillborn piglets per litter; MM: Number of mummified fetuses per litter; CEI: Cumulative expulsion interval; LW: Litter live-birth weight; BW: Birth weight; EI: Expulsion interval.

**Table 2:** Comparison of candidate binomial GLMM for stillborn piglets.

Model	Logit (p*_ij_*)	Fixed Effects								Random Effect	AIC	Final Model
		BW	BO	CEI	EI	BWCV	PG	LS	TF	Sow		
1		√	-	-	-	-	-	-	-	√	436.35	
2		√	√	-	-	-	-	-	-	√	416.81	
3		√	-	√	-	-	-	-	-	√	406.50	
4		√	-	-	√	-	-	-	-	√	438.25	
5	Stillborn	√	-	√	-	√	-	-	-	√	405.09	
6		√	-	√	-	√	√	-	-	√	405.34	√
7		√	-	√	-	√	√	√	-	√	406.19	
8		√	-	√	-	√	√	-	√	√	406.97	
9		√	-	-	-	√	√	-	-	√	411.47	
10		√	-	-	√	√	√	-	-	√	432.15	

BW: birth weight; CEI: cumulative expulsion interval; EI: expulsion interval; PG: parity group; BWCV: within-litter birth weight coefficient of variation; TF: time of farrowing; LS: litter size; AIC: Akaike Information Criterion. Model 6 was selected as the final model.

**Table 3:** Odds ratios for stillborn piglets from the final binomial GLMM.

Variable	Contrast/Reference	Odds Ratio	95% CI	*p*-Value
BW				
	0.0 kg increased (reference)	1	-	-
	0.1 kg increased	0.750	0.688–0.818	<0.001
	0.25 kg increased	0.487	0.392–0.605	<0.001
CEI				
	0 min increased (reference)	1	-	-
	60 min increased	1.711	1.395–2.156	<0.001
	120 min increased	2.929	1.902–4.512	<0.001
BWCV				
	0% increased (reference)	1	-	-
	Per 5% increased	1.228	0.978–1.542	0.077
	Per 10% increased	1.509	0.957–2.379	0.077
Sow parity				
	1 (reference)	1	-	-
	2	0.566	0.183–1.756	0.324
	3 to 5	1.184	0.407–3.445	0.757

BW: Birth weight; CEI: Cumulative expulsion interval; BWCV: within-litter birth weight coefficient of variation; CI: confidence interval.

## Data Availability

The data presented in this study is available on request from the corresponding author.

## Abbreviations

The following abbreviations are used in this manuscript:

AIC	Akaike Information Criterion
BO	Birth order
BW	Birth weight
BWCV	within-litter birth weight coefficient of variation
CEI	Cumulative expulsion interval
CI	Confidence interval
CO_2_	Carbon dioxide
CV	Coefficient of variation
EI	Expulsion interval
GLMM	Generalized linear mixed model
LS	litter size
OR	Odds ratio
PG	Parity group
PN	parity number
Q1–4	Quartile 1 to 4
TF	time of farrowing
